# *ACTN3* Allele Frequency in Humans Covaries with Global Latitudinal Gradient

**DOI:** 10.1371/journal.pone.0052282

**Published:** 2013-01-24

**Authors:** Scott M. Friedlander, Amanda L. Herrmann, Daniel P. Lowry, Emily R. Mepham, Monkol Lek, Kathryn N. North, Chris L. Organ

**Affiliations:** 1 Department of Ecology and Evolutionary Biology, Brown University, Providence, Rhode Island, United States of America; 2 Department of Earth and Environmental Sciences, University of Michigan, Ann Arbor, Michigan, United States of America; 3 Institute for Neuroscience and Muscle Research, The Children's Hospital at Westmead, Sydney, New South Wales, Australia; 4 Discipline of Paediatrics and Child Health, University of Sydney, Sydney, New South Wales, Australia; 5 Department of Anthropology, University of Utah, Salt Lake City, Utah, United States of America; 6 Department of Paleontology, Natural History Museum of Utah, University of Utah, Salt Lake City, Utah, United States of America; University of Cambridge, United Kingdom

## Abstract

A premature stop codon in *ACTN3* resulting in α-actinin-3 deficiency (the *ACTN3* 577XX genotype) is common in humans and reduces strength, muscle mass, and fast-twitch fiber diameter, but increases the metabolic efficiency of skeletal muscle. Linkage disequilibrium data suggest that the *ACTN3* R577X allele has undergone positive selection during human evolution. The allele has been hypothesized to be adaptive in environments with scarce resources where efficient muscle metabolism would be selected. Here we test this hypothesis by using recently developed comparative methods that account for evolutionary relatedness and gene flow among populations. We find evidence that the *ACTN3 577XX* genotype evolved in association with the global latitudinal gradient. Our results suggest that environmental variables related to latitudinal variation, such as species richness and mean annual temperature, may have influenced the adaptive evolution of *ACTN3 577XX* during recent human history.

## Introduction

Genes important for metabolism include the α-actinin family that code for contractile proteins in muscle [1]. In humans, two different genes code for skeletal muscle α-actinins: *ACTN2* is expressed in all skeletal muscle fibers, while *ACTN3* is expressed in fast-twitch muscle fibers. Mammalian *ACTN2* and *ACTN3* genes appear to have arisen from a gene duplication that occurred over 310 million years ago, before the divergence of birds and mammals [2]. Following the duplication of the ancestral *ACTN 2/3* gene, *ACTN3* evolved specific expression patterns in fast twitch muscle fibers, which are responsible for generating force at high velocity. Yet, approximately 18% of the human population is homozygous for an allele (R577X) that contains a premature stop codon in *ACTN3* [3], [4]. In the homozygous condition (577XX genotype) this leads to a complete deficiency of the α-actinin-3 protein, although α-actinin-2 partially compensates for the deficiency [5]. The absence of α-actinin-3 in humans has been shown to reduce strength, muscle mass, and fast-twitch fiber diameter, but increase skeletal muscle metabolic efficiency and resistance to fatigue [6]. Consequently, the *ACTN3* 577XX genotype has been hypothesized to be adaptive where efficient muscle metabolism is concerned, such as in environments with scarce resources or where endurance running has an impact on survival [7]. Together, these Darwinian hypotheses predict a significant evolutionary relationship of *ACTN3* 577XX genotype frequency and global latitudinal patterns of biodiversity. This relationship is predicted because factors related to food acquisition and hunting, such as species richness or mean annual temperature, show well-established global latitudinal patterns [8].

The *ACTN3* 577X substitution likely preceded the arrival of anatomically modern humans in Europe and Asia 40,000–60,000 years ago [9]. Since the evolution of modern humans, approximately 100,000–200,000 years ago, populations have occupied a wide range of habitats and adapted to use a variety of resources. Differences in environmental resources can drive even small genetic changes, such as single nucleotide polymorphisms, to high frequency in human populations. For example, using demographic HapMap data, allele frequencies related to subsistence have been found to change across different ecoregions [10]. It is therefore plausible that differences in the frequency of *ACTN3* 577XX may have arisen during the recent past, specifically in relation to the global latitudinal gradient. Here we test the *ACTN3* 577XX adaptation hypothesis using global genotype and biodiversity data, with Bayesian comparative methods that account for phylogeny and migration.

## Methods

*ACTN3* XX frequency data were obtained from the literature (Table 1). These data were derived from studies that compared the frequencies of the *ACTN3* polymorphism in elite athletes to the frequencies in control groups of the general population. We only used frequency data from the control groups (n = 3,351). *ACTN3* XX frequency data were Arcsin transformed before statistical analysis, a transform suitable for nominal and percentage data [11].

**Table 1:** Latitude, mean annual temperature, net primary productivity, and species richness data were obtained from the World Wildlife Fund Terrestrial Ecoregion Database [12].

Population	Latitude (Mean)	Temp (Mean, C^o^)	Ln Net Primary Productivity (g of carbon)	Ln Species Richness	XX frequency	XX Ref
Australian	26.42	20.47	11.25	6.07	10%	[30]
Bantu	27.96	18.44	11.27	6.29	1%	[30]
China	32.00	11.52	11.23	6.12	16.9%	[31]
Ethiopia	9.76	24.15	11.12	6.23	12%	[30]
Greece	40.00	12.06	11.39	5.86	18.3%	[32]
Israel	34.12	17.97	10.94	5.98	18%	[33]
Italy	42.61	11.13	11.50	5.78	21.57%	[34]
Japan	41.50	6.71	11.42	5.73	24%	[30]
Kenya	0.61	21.47	11.57	6.62	1%	[30]
Lithuania	47.66	6.35	11.49	5.82	10.4%	[35]
Nigeria (Yoruba)	7.96	25.34	11.49	6.41	0%	[30]
Papua New Guinea	6.61	24.99	11.68	5.74	15%	[30]
Russia	48.95	6.06	11.08	5.81	14.2%	[36]
Spain	40.64	12.62	11.36	5.8	19.9%	[30]
Sweden	61.33	3.63	11.04	5.65	25%	[37]

References for XX frequency are included in brackets after ACTN3 data.

Using the World Wildlife Fund Ecoregion database [12], we determined the average latitude, mean annual temperature (C^o^), net primary productivity (grams of carbon), and total species richness (tetrapods) data for each country/region. Although some of these metrics have changed over the time period in which *ACTN3* polymorphisms arose in human populations, they nonetheless show a consistent relationship with the global latitudinal gradient [13]. For example, the latitudinal biodiversity gradient (increasing numbers of species and higher taxa from the poles to the tropics) has been a striking large-scale pattern that extends back through the Mesozoic into the Paleozoic [14]. However, it should be noted that large climatic changes have occurred (e.g. glaciation and aridification of North Africa) over the time in which differences in *ACTN3* allele frequencies arose among human populations. Even so, we expect differences in our gene frequency data to have arisen relatively recently in line with modern climatic variation (as opposed to a novel mutation that may have had specific adaptive value in a past environment).

We curated our data by compiling a list of every ecoregion within each country. The list was constructed first using maps to form overestimated rectangles of minimum and maximum latitude and longitude around each country to account for irregular borders. Every ecoregion within these rectangles was gathered and narrowed down further using Google Earth (we limited Russia to west of Mongolia). By using the average latitudinal and longitudinal coordinates of each ecoregion, we were able to see if the ecoregion fell within the borders of the country in question. Ecoregions within the country were retained, while ecoregions outside of the country were removed from the list. One exception was Israel, which had no ecoregions within its borders, so the two closest ecoregions were used.

We produced a Bayesian posterior distribution of phylogenetic trees from whole mitochondrial sequences for the following human lineages: Aboriginal Australian (NCBI 134303155), Bantu (NCBI 160426755), Israeli (NCBI 145968101), Japanese (NCBI 61287226), Chinese (NCBI 292597164), Kenyan (NCBI 13272962), Papua New Guinean (NCBI 256946671), Lithuanian (NCBI 301017707) Russian (NCBI 290555741), Swedish (NCBI 215789486), Nigerian (EMBL AF346985), Italian (EMBL AF346988), Greek (EMBL GQ129165), Spanish (NCBI 74475826), and Ethiopian (NCBI 156459510). The whole mitochondrial genome of *Homo Neanderthalensis* (NCBI 196123578) was included as an outgroup.

The program MUSCLE [15] was used to align the genome sequences and a posterior distribution of trees was inferred with the program BayesPhylogenies [16]. BayesPhylogenies implements a reversible jump mixture model that fits more than one model of sequence evolution to the data without partitioning. We used a GTR model with gamma-distributed rate variation across sites (4 rates) on one chain for 20,000,000 iterations, sampling every 2,000 iterations. We also implemented the reversible-jump mixture model for pattern heterogeneity. We checked for convergence by examining a time-series plot for the log-likelihoods in Tracer [17].

Statistical tests that accounted for phylogeny (regression and ancestral character state reconstruction) were performed in the program BayesTraits [18] (http://www.evolution.rdg.ac.uk). BayesTraits accounts for confounding phylogenetic relatedness in continuous data using a generalized least square (PGLS) approach. It can also account for phylogenetic uncertainty by integrating analyses over more than one tree (or a distribution of trees). Before we ran the regression analysis, we tested whether a random walk model of character evolution or a directional model of evolution best fit our data. We found no evidence for directional evolution (highest Bayes factor = 1.8, see below). Our analysis also estimated phylogenetic signal (λ), a parameter that assesses the degree to which covariation among trait values follows the phylogeny during the random walk and regression analysis [18]–[21]. The Bayesian Markov Chain Monte Carlo (MCMC) settings in BayesTraits were as follows: 2,050,000 iterations with a sampling frequency of 100. The rate deviation setting (which determines acceptance of new proposals during the MCMC process) was set at 0.5 for the random walk test and 0.05 for the regression.

Significance was determined for the random walk test using Bayes Factors, calculated as two times the difference between the harmonic means of log-likelihoods for the two models. Bayes factors of three or more indicate positive evidence, five of more strong evidence, and 10 or more very strong evidence [22], [23]. Significance testing for the regression was determined by calculating the percent of the posterior distribution for β (the slope parameter) that fell outside of the null expectation (a slope equal to zero).

Statistical tests that account for both phylogeny and migration were performed in the statistical programming language R [24] using the package MCMCglmm [25], [26]. MCMCglmm can be used to fit generalized linear mixed models using Markov chain Monte Carlo techniques. Such models can account for non-independence of the data from population level dynamics (migration) and phylogenetic relatedness by including their matrices as random affect components. We followed the general procedure in Stone et al. [27] to construct the mixed model, using the program Migrate-n v3.2.16 [28] to generate a migration matrix. The migration matrix was generated using default settings with 150 full mitochondrial genome sequences (see Supplemental Information). For the migration matrix, the nearest positive definite matrix was determined with the R function nearPD (the script is available in the Supplemental Information).

## Results and Discussion

Recent work [29] has shown that genome-wide SNP data can be used to reliably reconstruct human phylogenetic/demographic history. Comparative statistical methods can now be used to analyze trait data within a species by accounting for both phylogenetic relatedness and for gene flow [25], [26]. In this manuscript, we build regression models that account for phylogenetic relatedness and gene flow to show that the *ACTN3* 577XX genotype may have evolved in association with global latitudinal patterns of biodiversity.

**Figure 1:**
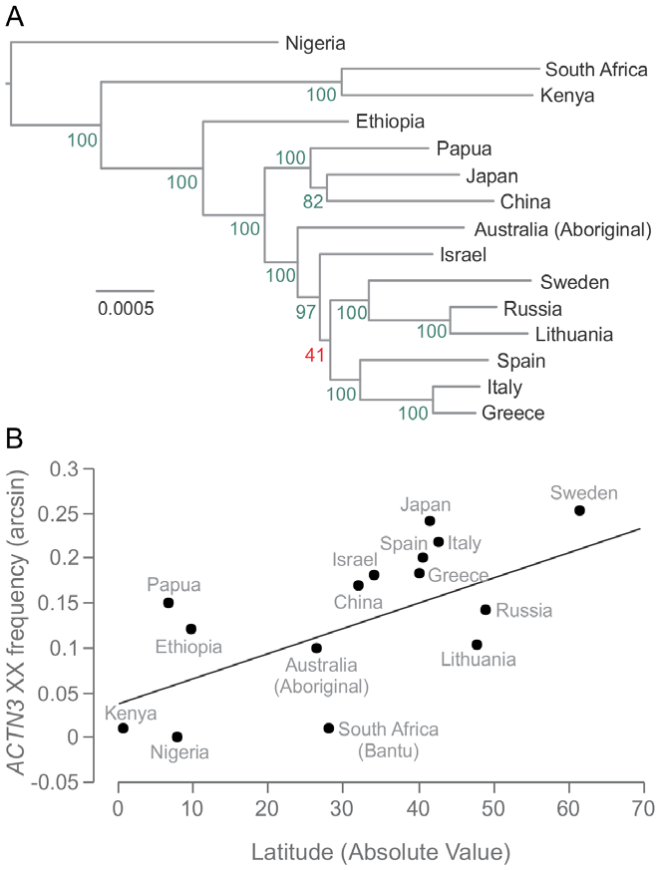
Human population phylogeny and relationship between *ACTN3* 577XX frequency and latitude. A, The Bayesian posterior consensus tree for the human populations used in this study (inferred from whole mitochondrial sequences). The numbers on the branches indicate the proportion of trees in the posterior distribution that contain a given clade. Branch lengths are in the number of changes per site. B, The relationship between *ACTN3* 577XX genotype frequency and latitude. The generalized linear mixed model that accounts for both phylogenetic relatedness and migration is arcsin(*ACTN3XX*) = 0.035–0.003 *Latitude (p-value for slope = 0.06).

We began by inferring a Bayesian posterior distribution of phylogenetic trees (Figure 1A), which matches the generally accepted demographic relationships among world-wide human populations [29]. Next we regressed *ACTN3* 577XX population frequency onto latitude, while estimating phylogenetic signal over the distribution of trees (and thereby accounting for phylogenetic uncertainty). We found strong phylogenetic signal in the regression model relating *ACTN3* 577XX frequency and latitude (maximum likelihood estimate of λ = 1). The model relating *ACTN3* 577XX frequency and latitude (absolute value) was supported, with a slope parameter for the generalized linear mixed model of 0.003 (lower 95% CI = 0.000, upper 95% CI = 0.005, p-value = 0.06). It should be noted here the importance of properly controlling for relatedness among the data – a standard linear regression model returns the same slope, but with an associated p-value of 0.006 and an R^2^ of 0.45. Note that goodness of fit is not reported in MCMCglmm, but an R^2^ of 0.32 is reported in BayesTraits, which controls for phylogeny, but not migration.

As discussed earlier, many factors covary with latitude that could explain *ACTN3* 577XX frequency variation. We tested only the most plausible factors to avoid overt testing of multiple hypotheses: species richness, mean annual temperature, and net primary productivity (natural log). We found no evidence for phylogenetic signal in the regression model relating *ACTN3* 577XX frequency and total vertebrate species richness (maximum likelihood estimate of λ = 0), but high levels of phylogenetic signal (maximum likelihood estimate of λ = 1) for both mean annual temperature and primary productivity. Accounting for phylogeny and migration, the model relating *ACTN3* 577XX frequency and total vertebrate species richness was supported, with a slope parameter of −0.23 (lower 95% CI = −0.36, upper 95% CI = −0.08, p-value = 0.016), as was the relationship between *ACTN3* 577XX frequency and mean annual temperature (slope of −0.006, lower 95% CI = −0.0126, upper 95% CI = 0.001, p-value = 0.09). However, we found no support for a model relating *ACTN3* 577XX frequency and net primary productivity (slope of −0.067, lower 95% CI = −0.31, upper 95% CI = 0.161, p-value = 0.56).

For present day latitude and associated data (such as species richness) to be meaningful in an evolutionary analysis, it must fairly represent latitudinal data of the past where genetic associations may have evolved. The relationship between latitudinal gradients and species richness, for example, is consistent through the recent past, with taxa preferentially originating in the tropics and spreading toward the poles [13], [14]. Moreover, the changes we analyze (differences in allele frequency) could have shifted relatively recently with respect to environmental change. However, it should be noted that we did not include marine species richness data in our analysis for coastal areas – and this could affect our species richness model. Moreover, although this study brings together disparate forms of data (which we view as a strength), the sample size for human populations was relatively small. Another limitation is the use of countries as demographic units, since countries vary in size, ecological diversity, as well as population size and heterogeneity (though our migration matrix should help account for this). For example, the 577X substitution is thought to have arisen in populations migrating out of Africa (Ethiopia) roughly 40,000–60,000 years ago [9]. Our analysis could, therefore, be confounded by including African population data, but 40,000 years is potentially enough time to allow for gene flow to increase the 577X allele frequency within African populations. Given the mechanical and physiological roles of *ACTN3* variation in the 577XX genotype, long-range linkage disequilibrium associated with *ACTN3* 577X (signature of positive selection) [6], and our results presented here, a neutral (demographically-driven) model of evolution is not supported. Available evidence instead suggests that changes in *ACTN3* 577XX frequency were driven by changing environmental conditions and resources associated with latitudinal migration.
